# Characterization of a Recovered Mediterranean Chicken Breed: The Case of Murciana

**DOI:** 10.3390/ani16121793

**Published:** 2026-06-10

**Authors:** Laura Martínez-Martínez, Achille Schiavone, Juan Orengo, Eva Armero

**Affiliations:** 1 Department of Agricultural Engineering, Universidad Politécnica de Cartagena Member of European University of Technology EUT+, Paseo Alfonso XIII 48, 30203 Cartagena, Spain; achille.schiavone@unito.it (A.S.); eva.armero@upct.es (E.A.); 2 Department of Veterinary Sciences, University of Turin, Largo Braccini 2, 10095 Grugliasco, TO, Italy; 3 Department of Animal Production, Faculty of Veterinary Science, University of Murcia, 30100 Murcia, Spain; jorengo@um.es

**Keywords:** chicken, local breed, conservation, breed valorization, dual-purpose

## Abstract

In recent decades, conservation of local poultry breeds has gained relevance to preserve genetic resources adapted to low-input systems and to enhance their valorization. This study addresses a key knowledge gap by providing a comprehensive characterization of the endangered Murciana chicken breed, native to southeastern Spain. We jointly evaluate recent population dynamics, conservation framework, morphology and morphometrics, growth patterns, and reproductive and productive traits. Data includes census and pedigree records, standardized morphological assessments, growth modeling, and production data from the conservation nucleus. The population increased from fewer than 150 registered animals in 2017 to more than 550 in 2024, indicating stabilization. The breed showed characteristics of slow-growing dual-purpose Mediterranean genotypes, with marked sexual dimorphism, Gompertz relative growth rates of 0.020 d^−1^ (males) and 0.023 d^−1^ (females), and adult weights of 3.2 kg and 2.4 kg, respectively. Carcass yield was moderate (61.9%), with higher leg (36.7%) than breast proportion (16.9%). Reproductive (fertility 88.6%, hatchability 80.6%) and laying performance (116.6 eggs/hen/year) were consistent with local extensive systems. These results provide a robust baseline to support conservation, genetic management, and sustainable use of the Murciana chicken breed, contributing to its long-term preservation and valorization.

## Simple Summary

Local poultry breeds are important for sustainable farming and biodiversity, but many are at risk and poorly studied. This work focuses on the Murciana, a native chicken breed from southeastern Spain that is currently endangered. The aim is to describe its population trend, main physical features, and productive performance. Results show that the population has increased in recent years, indicating successful recovery efforts. The breed shows moderate growth, body size and egg production levels, suitable for low-input farming systems. Overall, the Murciana chicken breed appears to be a useful local resource with potential for conservation and sustainable management.

## 1. Introduction

The global poultry sector has undergone rapid intensification in recent decades, driven by genetic improvement, advances in nutrition, and the widespread adoption of highly specialized commercial lines [1,2,3]. Although this model has markedly increased production efficiency, it has also accelerated the erosion of genetic diversity among domestic chicken populations, particularly affecting traditional breeds maintained under smallholder or low-input systems [4,5]. In this context, local breeds represent a strategic reservoir of genetic diversity, contributing traits related to rusticity, adaptation to harsh environments, suitability for extensive production systems, and differentiated product-quality attributes [6,7]. These characteristics are gaining relevance under current challenges associated with climatic variability, resource limitation, and the need for more sustainable poultry production models [8].

This perspective is reinforced by recent international and European policy frameworks. The European Green Deal, through initiatives such as EU Biodiversity Strategy and the Farm to Fork strategy, together with the United Nations Sustainable Development Goals (SDG 2: Zero Hunger, and SDG 15: Life on Land), emphasize the preservation of animal genetic resources and the promoting of resilient, low-input agri-food systems. However, despite these favorable policy environments, many native chicken breeds still lack essential scientific information regarding their population structure, phenotypic characteristics, and productive performance [9,10]. This knowledge gap hinders the effective design of conservation, breeding, and valorization strategies, thus limiting the integration of these genetic resources into sustainable production systems.

In Spain, the National Program for the Conservation, Improvement and Promotion of Livestock Breeds was established in 2008 to safeguard and develop national animal genetic resources (Royal Decree 45/2019). This program includes the Official Catalog of Spanish Livestock Breeds, which currently lists 22 native avian breeds (20 chicken breeds and two goose breeds), all classified as threatened. Although conservation frameworks are in place and several of these avian breeds are included in official breeding programs (15 of 22) [11], comprehensive characterizations remain scarce. Within this context, the Murciana chicken breed, native to the southeastern region of the Iberian Peninsula, constitutes a paradigmatic case of a local breed with high potential but limited available information.

The Murciana chicken breed was firstly described in the early twentieth century [12], later declared disappeared in the late 1980s [13], and subsequently recovered following the rediscovery of remnant populations during the 1990s [14]. Since then, recovery initiatives and the establishment of the breed association Amigos por la Gallina Murciana—AGAMUR (Friends of the Murciana chicken breed)—have played a key role in preserving the breed and maintaining its genetic identity, supported by an official herdbook (Decree 129/4 June 2010) that establishes animal registration procedures and defines the official breed standard. Currently, the Murciana chicken breed is officially recognized as endangered (Order APA/3628/2007), and is predominantly raised under extensive management conditions, representing both a genetic and cultural heritage linked to Mediterranean agroecosystems. Conservation efforts are structured around controlled mating schemes aimed at preserving genetic variability and maintaining reference populations for breed conservation, with selection and mating decision increasingly supported by genetic information [15]. In parallel, recent initiatives have focused on breed valorization, including the “100% Murciana Autochthonous Breed” (Royal Decree 505/2023) quality label in 2023 and the implementation of projects such as ECOGAMUR, which evaluated extensive production systems and the use of alternative feed ingredients, or GENEGAMUR which currently addresses the breed conservation through alternative product valorization.

The Murciana is a Mediterranean-type, dual-purpose (meat–egg) breed, has a wheaten coloration, with two varieties (silver and salmon), and is traditionally characterized by rusticity, adaptability to warm environments, and robustness under low-input conditions [14,16]. Despite its relevance, available information regarding its population status, morphological variability, growth patterns, and productive and reproductive performance remains limited, fragmented and often outdated. Existing references are largely restricted to historical descriptions, local dissemination documents, or national journals, and data integrating these aspects are still lacking [17,18].

Therefore, updated and robust data on the Murciana chicken breed are essential not only to support conservation and breeding strategies but also to assess its potential and functional relevance within sustainable poultry systems. Accordingly, the present study aims to provide a comprehensive characterization of the Murciana breed by: (i) evaluating its current population structure and geographical distribution; (ii) describing its morphological and morphometric traits; and (iii) analyzing its productive and reproductive performance under current management conditions.

## 2. Materials and Methods

### 2.1. Ethical Statement

Breed characterization was conducted at the Tomás Ferro Experimental Farm installations, belonging to the Technical University of Cartagena (UPCT), Spain. The experimental protocol was approved by the Ethical Committee of the Technical University of Cartagena (expedient n° CEI23_004). No action involving pain or suffering was practiced.

### 2.2. Conservation Framework and Population Data

The conservation program of the Murciana breed is coordinated by AGAMUR and supported by UPCT, under the framework of the Conservation and Improvement Program for the Murciana chicken breed [18]. The program integrates in situ conservation through the network of associated farmers and controlled breeding at the experimental station. Standardized management and recording protocols are implemented across participating farms under the supervision of the breed association.

Population census information was obtained, whenever available, from the Spanish National System on Zootechnical Resources (ARCA, Spain) and cross-checked with the official herdbook of the breed for the period 2017–2024. Census data were analyzed according to the categories established in the Murciana herdbook: foundation register (FR), including the initial individuals used to establish the herdbook; basic section (BS), comprising animals registered at birth that met minimum genealogical criteria; breeding stock with merits (MB), including breeders that demonstrated superior performance within the program; annex section (AS), including birds excluded from the previous categories due to the absence of parental registration; total breeding stock (TB), consisting of animals evaluated and approved for reproduction; and total animals (TA), defined as the sum of FR, BS, MB, and AS. In addition, the number of farmers contributing records to the herdbook in each year was evaluated. For the purposes of the study, these farmers were defined as “active farmers” and included only association members who provided annual herdbook records, rather than the total number of farmers associated with the breed.

Data on total farmers, their characteristics, and geographical distribution patterns, up to 2024, and overall data on the conservation status were obtained in collaboration with the association AGAMUR and the Tomás Ferro Experimental Farm, respectively. The geographical location of each registered facility hosting the Murciana chicken breed was compiled from the association registry.

### 2.3. Birds and Husbandry

**Figure 1:**
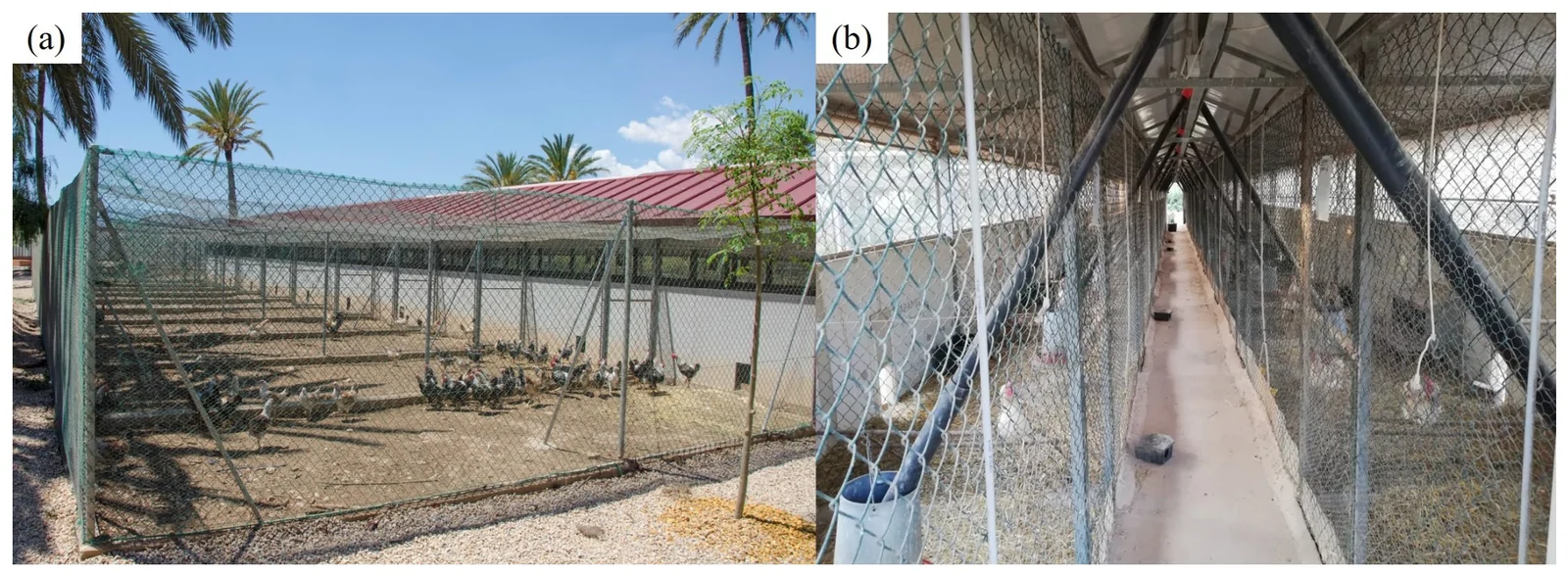
Outdoor (**a**) and indoor (**b**) of facilities of the Tomás Ferro Experimental Farm (UPCT).

Focusing on the characterization of the Murciana breed, the animals analyzed were selected from the conservation and breeding nucleus managed by Tomás Ferro Experimental Farm (Figure 1), including both adult breeding birds (hereafter referred to as breeders), and young birds (hereafter referred to as growing birds).

For morphological, morphometric, and growth analysis, eggs from 16 breeder groups (each composed of one male and 5–6 females) were artificially incubated, and a total of 125 chicks (71 males and 54 females) from three hatching batches produced in 2023 (≈40 chicks per batch) were used for the main characterization. Chicks were monitored from hatching until 133 days of age. Moreover, 20 males were selected from the last batch at 133 days for carcass quality assessment. In addition, all the breeders of the farm were included to assess the morphological standard of the breed and morphometric parameters: 12 males (660 days old on average) and 45 females (392 days old on average).

During the first weeks of life, chicks were housed in an indoor brooding enclosure under controlled temperature and lighting conditions provided by a 250 W infrared lamp. Chicks were reared in brooding units consisting of enclosed boxes (1.2 m^2^) equipped with a heat source and connected to an adjacent compartment (2 m^2^), allowing chicks to move freely within the unit. Subsequently, birds were transferred to modules on the farm, where they remained under natural lighting, temperature, and ventilation conditions until the end of the study. Each module consisted of a covered area of 5 m^2^ (≈0.13 m^2^/bird) equipped with feeders and drinkers and connected to an uncovered outdoor area of 24 m^2^ (≈0.6 m^2^/bird). The outdoor area was fenced and covered with bird-proof netting. From three months old onwards, males and females were reared in separated modules. All birds had free access to water and were fed ad libitum with diets formulated and supplied by a local company. Growing birds received a commercial feeding program designed for their own slow-growing commercial lines, consisting of four consecutive phases. According to the manufacturer, crude protein levels ranged from approximately 21–22% in the starter phase to 17–18% in the final phase, while metabolizable energy increased from about 2850 to 3050 kcal/kg.

The breeders were located on the same farm and maintained under the same housing conditions described above, with the only difference being that breeders were fed a specific breeder diet, formulated by the same company specifically for the Murciana breeding stock, containing approximately 15–16% crude protein, 2800 kcal/kg metabolizable energy, 2.9–3.0% of calcium, and 0.36% of available phosphorus.

### 2.4. Reproductive Performance

Fertility and hatchability were calculated separately for each breeding pen within each of the three incubation batches conducted during the 2023 breeding season. Each batch included eggs from nine breeding pens, resulting in a total of twenty-seven pen-level records. Eggs were collected daily, recording the pen number, and stored in a natural-environment room for up to 7 days. Once 60 suitable eggs were accumulated, they were artificially incubated in an automatic incubator (Ova-Easy 100 Advance EX, Brinsea, Titusville, FL, USA). Incubation was carried out for the first 19 days at 37.7 ± 0.1 °C, 50% relative humidity, with automatic turning every 45 min. On day 19, egg turning stopped, trays were covered, and relative humidity was increased to 60%. Incubation ended on day 23, when non-hatched eggs were examined and classified as infertile or showing embryonic mortality. The fertility rate was calculated as the proportion of fertile eggs relative to the total number of incubated eggs, whereas hatchability was expressed as the proportion of chicks hatched from the total number of fertile eggs.

### 2.5. Growth Performance

Mortality and the presence of clinical signs indicative of disease were recorded daily throughout the experimental period. Growth performance was evaluated separately for males and females. Individual body weight (BW) was recorded at 15-day intervals from hatching to 133 days of age, and average daily gain (ADG) was calculated accordingly.

The BW data were used to model growth trajectories using the Gompertz nonlinear function up to 126 days old [19]:

(1) $$Y=A*exp(-b*\exp\left(-k*t\right))$$

where *Y* (g) is the BW reached at age *t* (d); A represents the asymptotic BW (BWa, g); b is a scaling parameter related to the initial growth phase; and *k* is the maturation rate constant (d^−1^).

Derived growth traits were calculated from the fitted model referring to the inflection point (ip), including age at ip (Tip = (ln(b))/k, d), BW at the ip (BWip = A/e, g), and maximum growth rate (MGR = A·k/e, g/d). The goodness of fit was assessed using the coefficient of determination (R^2^).

### 2.6. Morphological Characterization

External morphological traits of growing birds were described visually following the official breed standard (Decree 129/4 June 2010). Each animal was scored on a scale from 1 to 10 for the following morphological groupings: head; tail; chest, abdomen and back; thighs, shanks and toes; comb, wattles and ear lobes; and plumage color. To calculate the total phenotypic score (maximum 100 points), individual traits scores were weighted according to the breed standard: head was assigned a weighting factor of 2, plumage color of 4, and the remaining traits of 1.

Photographs of representative males and females were taken at chick (15 days old), growing birds (133 days old), and breeders (male: 350 days old; female: 454 days old), to illustrate plumage color and morphological traits of each stage.

### 2.7. Morphometric Measurements

Morphometric traits were measured according to FAO guidelines [20] to quantify body structure and developmental patterns. Measurements were collected at two physiological stages, growing and breeder, and included BW (g), body length (BL, cm), thoracic circumference (TC, cm), tarsus diameter (TD, cm), tarsus length (TL, cm), wingspan (WS, cm), and wing length (WL, cm).

### 2.8. Carcass Evaluation

Birds were slaughtered at the facilities of a local company, following the company standard protocol. Stunning was performed using a multi-phase carbon dioxide system, in which birds were exposed to a CO_2_ supply tank with gradually increasing concentrations of 20%, 30%, 35%, 40%, and 60%, for 60 s at each stage. The slaughter, performed via neck incision, and the pluck were carried out and supervised by qualified company personnel. Evisceration was carried out manually by qualified personnel of the UPCT, maintaining the head, feet, heart, liver, and gizzard, designated as Brute Carcass (BC). Post evisceration and 24 h cooling at 4 °C, BC was weighed (BCW). The BCW was then cut according to the method described by Jensen [21]. Abdominal fat was collected during evisceration as the fat is easily removable from the visceral package. Mesenteric fat was not included. Residual fat from carcass dissection was negligible and not systematically recorded. Therefore, the variable represents dissectible abdominal fat. Head, feet, heart, liver, and gizzard were removed and weighed individually, and the remaining carcass was considered as Net Carcass (NC), which was also weighed (NCW). BC and NC yields (BCY and NCY), as well abdominal fat percentage, were expressed relative to slaughter weight (SW). Breast, breast skin, both leg quarters and wings, were subsequently removed and weighed individually. The remainder carcass frame was collected and weighed, and all component yields are expressed relative to both BCW and NCW.

### 2.9. Egg Production

Egg production was recorded at pen level under conservation management conditions over the study period (January to December 2023). Eggs were collected daily in each pen, and both the total number of eggs and the number of hens present in each pen were registered. Annual egg production per hen was estimated from 10 pens (mean: six hens per pen; 1.6 years of age), based on the ratio between total egg output and cumulative hen days. Seasonal variation in laying performance was evaluated using a subset of pens (*n* = 6) that maintained a constant number of hens between April and September, thereby minimizing the confounding effects of flock renewal. Pens were grouped according to average hen age (one and two years). Egg production was aggregated into biweekly periods. Laying intensity (HDEP, %) was expressed as hen-day egg production, calculated as the ratio of the number of eggs produced to the total number of hen days within each period, multiplied by 100. For each age group and period, mean HDEP values were calculated, while individual pen trajectories were retained to illustrate within-group variability.

### 2.10. Statistical Analysis

Population census, geographical distribution, and facility-related data were summarized using descriptive statistics. Reproductive performance, morphological and morphometric traits, growth parameters, and carcass traits data are presented as mean ± standard deviation (SD). Growth curves were modeled using nonlinear mixed-effects models based on the Gompertz function. Sex and batch were included as fixed effects on all model parameters (A, k, and b), while individual animal was included as a random effect on A and k parameters.

To describe the geographical distribution of the breed, spatial data were processed and visualized using QGIS (version 3.34; QGIS Development Team, Zürich, Switzerland, 2024). All statistical analyses were performed using R software (version 4.3.3), and figures were generated using R software (version 4.5.2; R Development Core Team, Vienna, Austria) and Python (version 3.12.4; Python software Foundation, Beaverton, OR, USA).

## 3. Results

### 3.1. Population Structure and Geographical Distribution

**Figure 2:**
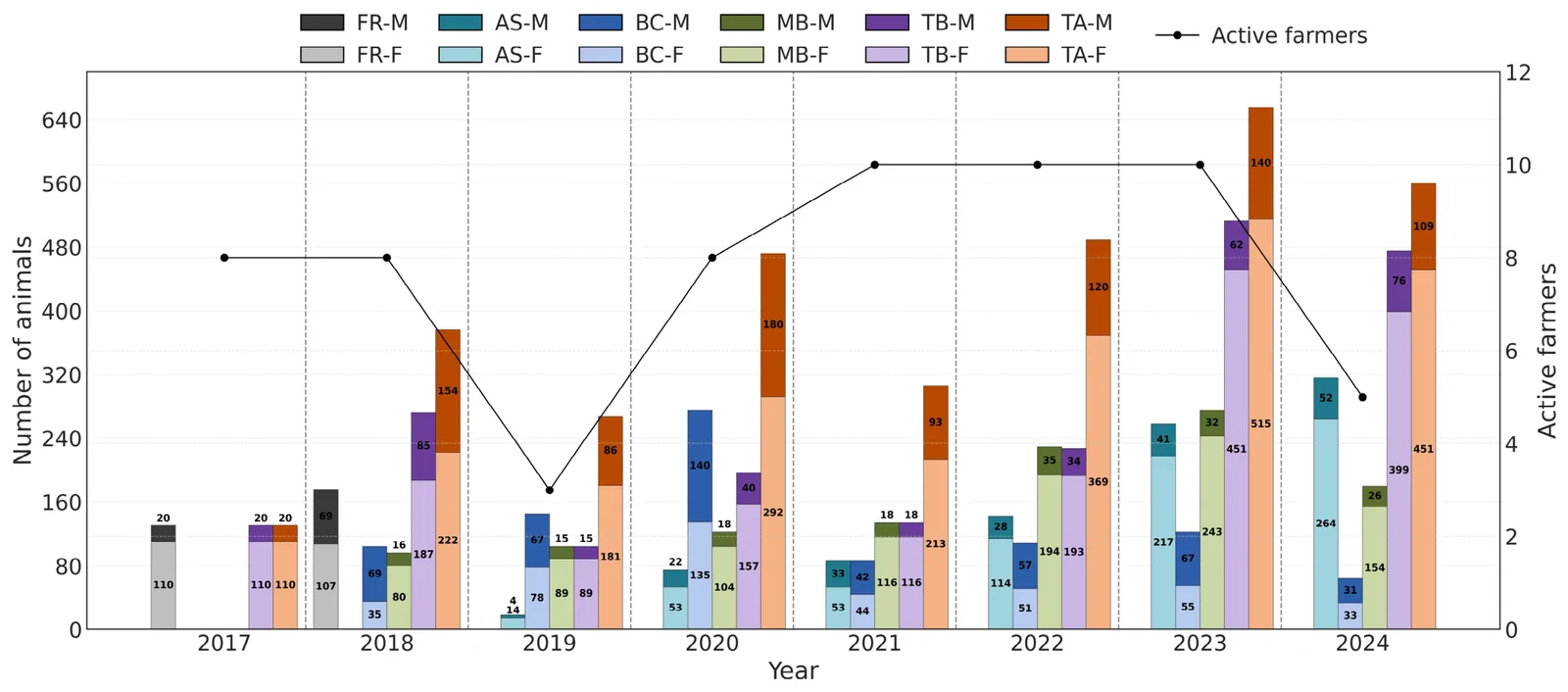
Evolution of the number of active farmers and the census recorded in the herdbook from 2017 to 2024. Census data are presented according to breeding categories and sex: foundational register (FR); annex section (AS); basic section (BS); breeders with merits (MB); total breeding stock (TB); total animals (TA); males (M); females (F).

During the 2017–2024 period, the total number of Murciana chickens registered in the herdbook increased from 130 to 560 individuals, reaching a maximum of 655 animals in 2023 (Figure 2). At the beginning of the study period, the census consisted of 110 females and 20 males forming the foundation register, which remained active until 2019. By 2024, a total of 451 females and 109 males were registered across the different categories, of which approximately 80% belonged to the TB category. From 2019 onwards, a progressive increase was observed in the number of animals included in BS and MB categories, as well as in the total population. Overall, the data indicate sustained growth of both TB and TA populations until 2023, followed by a slight contraction in the final year of the study period. This trend reflects adjustments in the census structure and reproductive dynamics, with a current breeder female-to-male ratio of 5.3, compared with a 7.2 in 2023. In parallel, the number of active farmers ranged from three to 10 farmers throughout the study period, with five farmers recorded in 2024.

In 2024, a total of 37 farmers associated with the Murciana breed were recorded (Table 1). Of these, six held an official farm code in the General Registry of Livestock Farms (REGA, Spain), one farmer was certified under the “100% Murciana Autochthonous Breed” quality label, and three restaurants held the same certification. Most farmers (35) used the breed primarily for self-consumption, whereas only one operated commercially and one corresponded to a selection and conservation nucleus officially classified as a multiplier farm (that is, UPCT). The farmer population was predominantly male, with 34 men and three women, representing a female participation of 8.1%.

**Figure 3:**
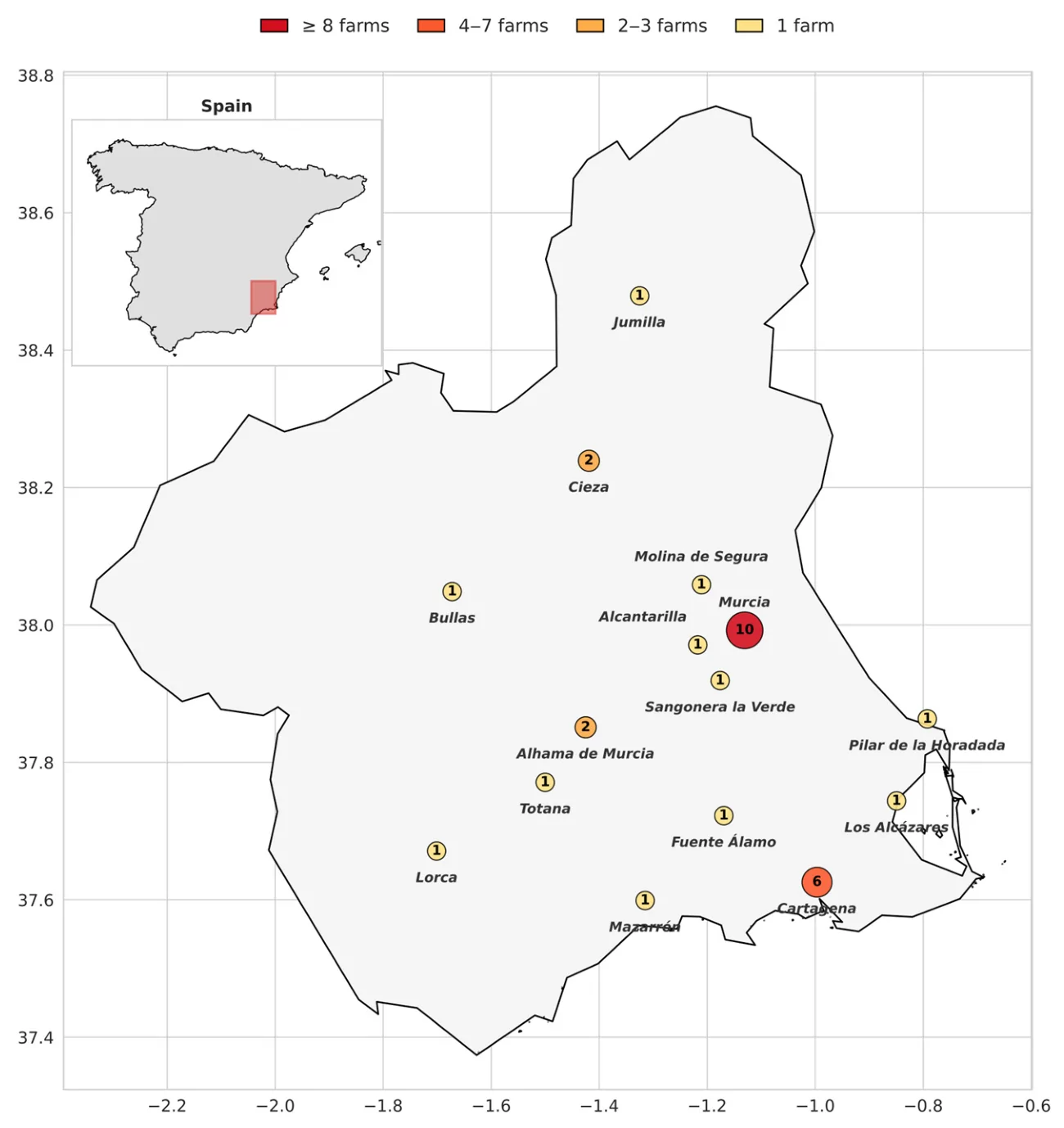
Geographical distribution of Murciana chicken breed farms across municipalities in the Region of Murcia in 2024.

The geographical distribution of farmers and their facilities was concentrated mainly in the Region of Murcia (Figure 3), with the highest number of farms located in the municipality of Murcia (10), followed by Cartagena (6). Fewer facilities were identified in the municipalities of Alhama de Murcia, Cieza, Bullas, Lorca, Totana, Fuente Álamo, and Pilar de la Horadada, the latter located in the province of Alicante.

### 3.2. Morphological Characterization and Morphometric Traits

**Figure 4:**
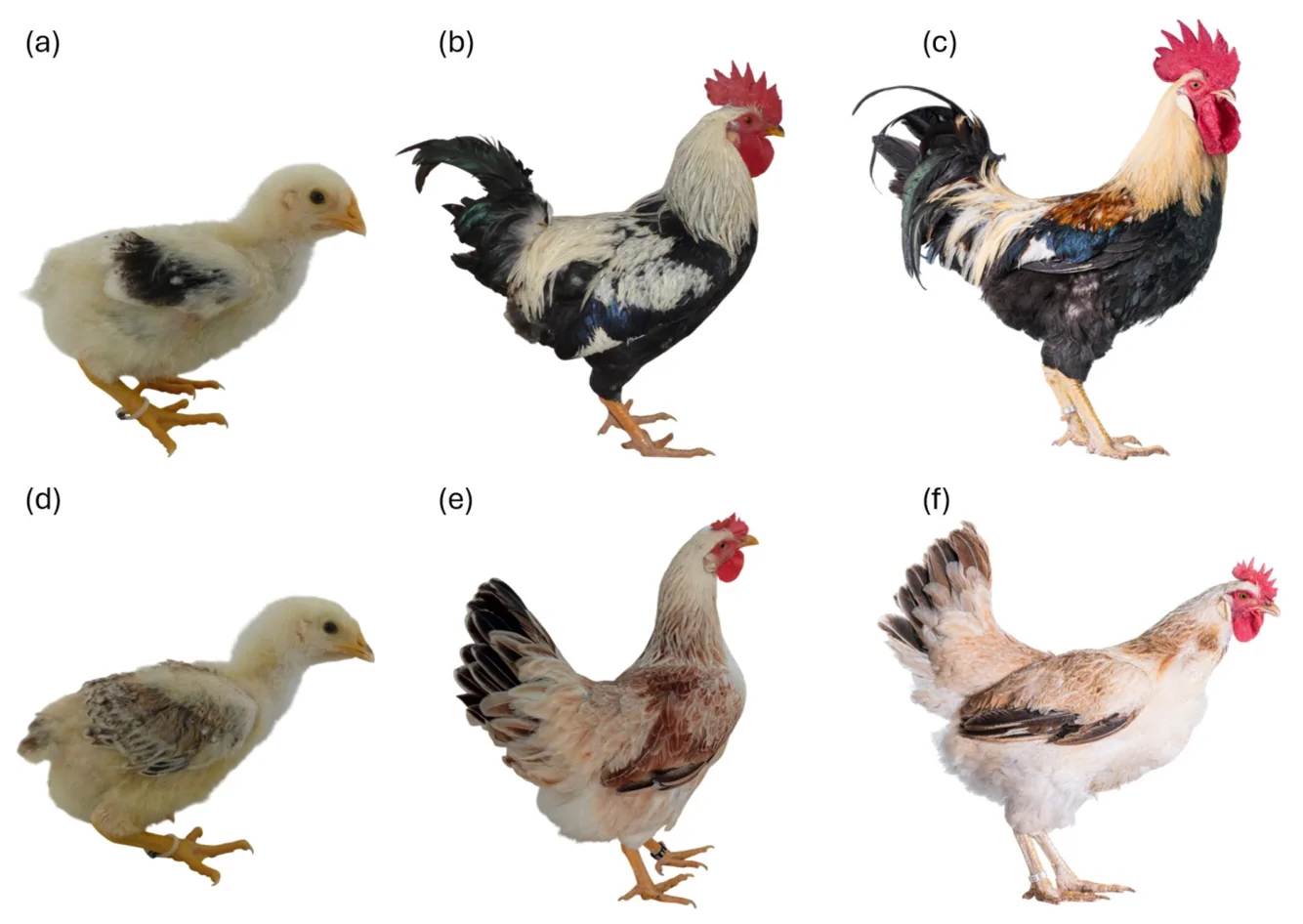
Morphological traits of Murciana chicken breed: (**a**) male chick (15 days old); (**b**) growing male (133 days old); (**c**) breeder male (350 days old); (**d**) female chick (15 days old); (**e**) growing female (133 days old); (**f**) breeder female (454 days old).

The main morphological traits of the Murciana chicken breed were described in males and females at different developmental stages (Figure 4). At hatching, chicks of both sexes exhibited yellow down, whereas as definitive plumage developed, females showed predominantly brown coloration, while males developed mainly black plumage, allowing visual sex differentiation from approximately 15 days of age. The growing male (Figure 4b) exhibited the phenotype corresponding to the silver variety, while breeder male (Figure 4c) showed the salmon variety. In both varieties, males presented black chest and thighs, black tail feathers with greenish reflections, black primary feathers with white external remiges, and cream, whitish neck and saddle feathers. The comb was single, large and erect, with 5–7 points, and ear lobes were white. The main morphological difference between varieties was observed in shoulder and back plumage, which was reddish-orange in the salmon variety and uniformly white in the silver variety.

Females (Figure 4e,f) were characterized by cream-whitish chest and thighs, brown-toned neck feathers, and a uniformly brown back and shoulders with slightly lighter edging. Ear lobes were white, and the comb was single, with a tendency to fall slightly to one side. Differences between varieties in females were limited to variations in coloration intensity. The growing female showed a coloration pattern similar to that of the breeder female, whereas the young male displayed a more immature morphology than the breeder male, with incomplete development of the tail and dorsal plumage.

The standard morphological evaluation scores of growing Murciana males and females are shown in Table 2. Mean total scores were 86.5 ± 4.45 in males (range: 77–96) and 90.2 ± 3.93 in females (range: 81–96). For both sexes, most evaluated traits showed mean values above 8 points. The highest scores were recorded for head and tail traits, whereas greater variability was observed for comb, wattles, ear lobes and coloration traits. Females showed slightly higher mean scores than males across most evaluated parameters, particularly for chest, abdomen and back, and for comb, wattles and ear lobes.

Morphometric measurements of Murciana chickens according to sex and age group are presented in Table 3. BW increased from the growing to the breeder stage in both sexes, from 1820 to 2448 g in females and from 2717 to 3197 g in males, corresponding to relative increases of 34.5% and 17.7%, respectively. Among linear body measurements, BL, TL, WS and WL showed similar mean values between growing and breeder birds within each sex. TC was higher in breeder than in growing birds, increasing by 3.8% in females and 4.1% in males. TD was similar between growing and breeder females, whereas breeder males showed a higher mean value than growing males.

### 3.3. Growth Performance

Observed BW and ADG are presented in Table 4. The BW increased progressively with age in both sexes, with males consistently showing higher values than females throughout the entire rearing period. At 126 days of age, males reached 2655 ± 249 g, whereas females reached 1772 ± 217 g. The ADG increased with age in both sexes up to 42–56 days, reaching maximum values of 26.1 ± 3.87 g/d in males and 20.4 ± 3.61 g/d in females, and decreased thereafter. Over the entire growing period (0–126 days), males showed higher ADG than females (20.2 ± 1.93 vs. 13.4 ± 2.06 g/d).

**Figure 5:**
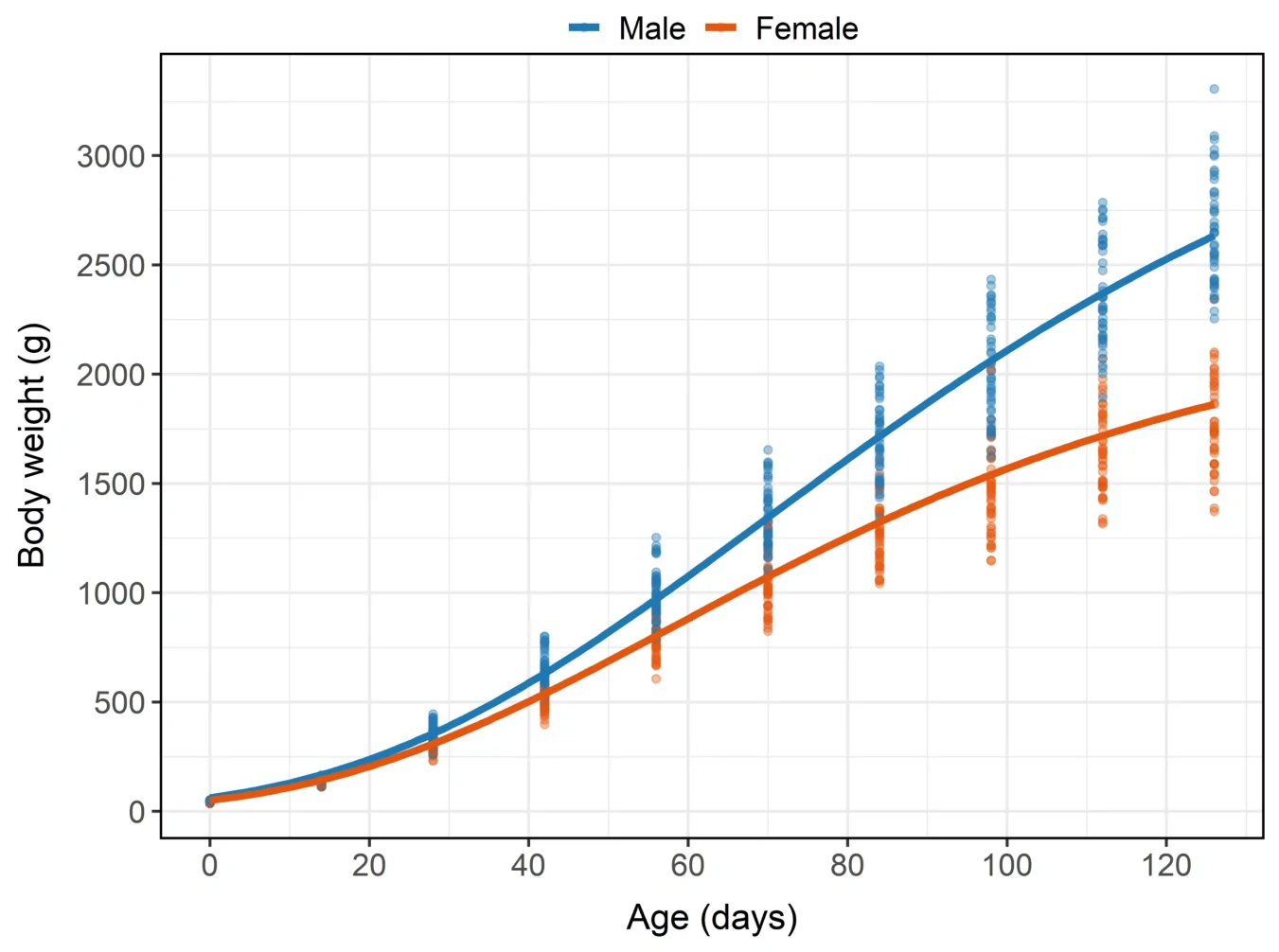
Sex-specific growth trajectories of Murciana chickens described by a Gompertz nonlinear mixed-effects model. Solid lines represent model-predicted body weight based on fixed effects (averaged across batches), and points correspond to observed individual measurements (males, *n* = 71; females, *n* = 54).

Growth curves were adequately fitted by the Gompertz model (Figure 5; R^2^ = 0.997). Parameters are shown in Table 5, males exhibited a higher BWa (3704 g) than females (2317 g), the k parameter was slightly higher in females (0.023 d^−1^) than in males (0.020 d^−1^), the Tip was 70.4 days in males and 58.2 days in females, with corresponding BWip values of 1363 and 852 g, therefore MGR was higher in males (27.4 g/d) than in females (19.8 g/d).

### 3.4. Reproductive and Productive Performance

**Figure 6:**
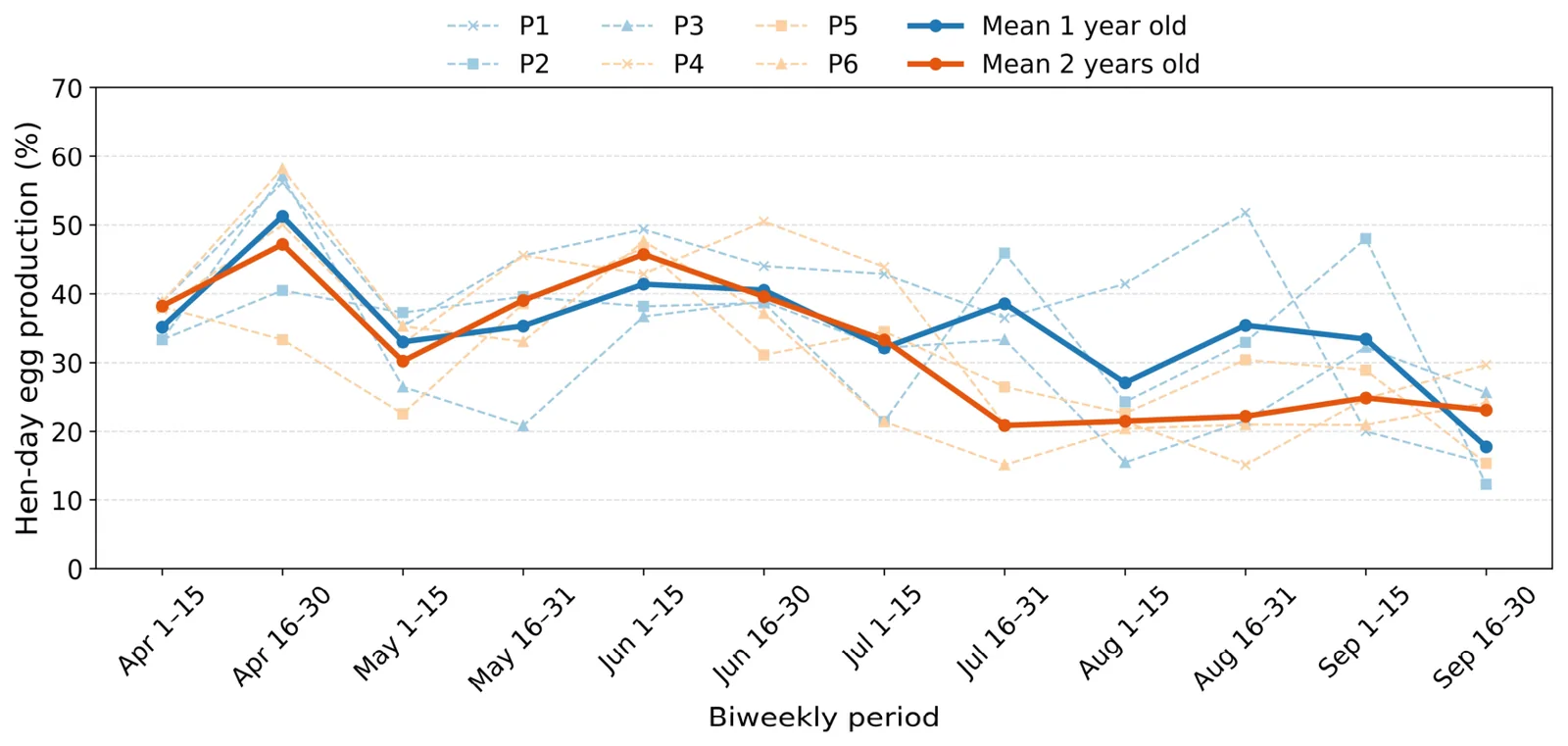
Seasonal variation in laying intensity (HDEP, %) by hen age. Biweekly HDEP from April to September in one- and two-year-old hens. Solid lines indicate group means, whereas dashed lines show individual pens (*n* = 3 pens per group).

Across the 27 pen-level records, mean fertility of incubated eggs was 88.6% (range: 66.7–100%), whereas mean hatchability reached 80.6% (range: 25–100%). Annual egg production averaged 116.6 eggs per hen per year. Seasonal variation in HDEP is shown in Figure 6. In one-year-old hens, HDEP reached ≈50% in April and decreased towards summer, with minimum values in July–August, followed by a slight increase in late summer and a decrease in September. In two-year-old hens, HDEP ranged from ≈40–46% in spring to ≈20–22% in summer, following a similar temporal trend. Within each age group, variability among pens was evident across periods, particularly during transitional phases, as indicated by the dispersion of individual trajectories.

Carcass characteristics of Murciana growing males are presented in Table 6. The SW was 2730 ± 143 g, with BCW and NCW of 2117 ± 115 g and 1688 ± 95.9 g, corresponding to yields of 77.6 ± 2.57% and 61.9 ± 2.57%, respectively. Leg quarters represented the main commercial cut, accounting for the highest proportion of both BCW (29.3 ± 1.51%) and NCW (36.7 ± 1.86%), followed by breast and wings. Whole breast yield reached 16.9 ± 1.3% of NCW, while wings accounted for 12.4 ± 0.78%. Abdominal fat deposition was low (1.20 ± 0.87% of SW). Internal organs showed limited contribution to carcass weight (<3% of BCW individually), whereas non-edible parts (feet, head, and neck) together accounted for approximately 15% of BCW.

## 4. Discussion

### 4.1. Population, Farmers Dynamics, and Breed Initiatives

The Murciana chicken breed experienced a sustained increase in census size between 2017 and 2024, rising from 130 to 560 registered individuals. This trend indicates a transition from an initial recovery phase to a more consolidated stage of conservation and reflects the effectiveness of the strategies implemented within the official conservation program. Similar census trajectories have been reported for other Spanish local chicken breeds under structured conservation schemes, such as the Utrerana chicken breed, where progressive increases in both registered animals and active farmers have been documented [22]. Despite this growth, the Murciana chicken breed remains classified as “endangered–maintained” according to FAO criteria [23,24], as its population size is still limited but supported by an officially recognized conservation program. In the Spanish context, the Murciana occupies an intermediate position among local chicken breeds, with a census size well below that of more consolidated populations such as Castellana Negra or Gallina del Sobrarbe (>1400–2200 animals), but above other endangered chicken breeds such as Empordanesa, Prat or Mallorquina (<350 animals) [11]. Comparable census sizes have been reported for several European local chicken breeds classified as at risk, whose long-term viability depends on continued institutional support and structured genetic management [25,26]. In this context, the Murciana fits the general pattern described for European local poultry populations: small census size, recent growth linked to conservation initiatives, and potential vulnerability to genetic drift if effective population size and pedigree control are not reinforced [5,27,28,29], which highlights the importance of consolidating long-term genetic management strategies and controlled breeding plans [15,30,31].

The farmer network of the Murciana chicken is dominated by small-scale holdings primarily oriented toward self-consumption, with only one farmer clearly engaged in commercial production. This structure closely resembles that reported for other Spanish and Mediterranean local chicken breeds, where flocks are typically small and maintained outside intensive market-oriented systems [22]. The low participation of women among Murciana breeders is consistent with observations reported for several local breed associations in developed countries, where the involvement of women and younger generations has been identified as a key factor for the diversification of production models and generational renewal [28,32]. Despite the growing number of farmers associated with the Murciana breed, only a proportion of holdings are currently fully registered in the REGA. This situation reflects a transitional phase commonly described in local breed conservation programs, in which small-scale and self-consumption-oriented farmers progressively formalize their administrative status as conservation initiatives consolidate and institutional support increases [33]. In parallel, participation in the herdbook remains partial among farmers primarily oriented towards self-consumption. In such systems, animals are frequently maintained outside formal commercial circuits, reducing the perceived need for individual registration, a pattern also reported for other local breeds in Europe [28].

From a territorial perspective, the strong geographical concentration of farmers within a limited area increases vulnerability to sanitary events, movement restrictions or environmental disturbances. This risk has been repeatedly highlighted in FAO guidelines for small–conserved populations and underlines the importance of gradually expanding the number of formally registered holdings and diversifying farmer locations to improve the resilience and long-term sustainability of the conservation program [23,34].

The current status of the Murciana chicken breed cannot be understood without the coordinated action of the conservation nucleus, the breed association and institutional support. This organizational framework is consistent with other European conservation programs in which a reference nucleus maintains animals with high breed standards, reliable pedigree information and detailed records, while farmer associations facilitate herdbook registration and dissemination of the breed standard [28,35]. The granting of the “100% Murciana Autochthonous Breed” quality label and the implementation of initiatives such as ECOGAMUR and GENEGAMUR place the Murciana chicken breed within the paradigm of “conservation through use”, which has been identified as a key strategy for ensuring the long-term sustainability of local breeds with moderate productive performance [34,36]. However, commercial adoption of this label remains limited compared to other well-documented cases, such as the Mos chicken in north-western Spain or the Poulet de Bresse in France, where product differentiation has generated stable niche markets and added economic value [37,38].

Experiences reported in Italy, Portugal and Spain indicate that the consolidation of local chicken breeds is often associated with short marketing chains and an explicit linkage between product, territory and gastronomy [39,40,41,42]. Within this framework, the potential of the Murciana chicken breed in terms of commercial valorization and product differentiation remains underexploited, highlighting clear opportunities for economic development strategies complementary to genetic conservation actions.

### 4.2. Morphologic and Morphometric Profile

The morphological profile of the Murciana chicken corresponds to a light Mediterranean type, characterized by moderate body size, and marked sexual dimorphism [17]. The clear differentiation between plumage varieties in males, contrasted with more homogeneous female phenotypes, is consistent with observations in other Mediterranean and Iberian chicken breeds, where plumage polymorphism is more pronounced in males due to sexual selection and farmer preferences [35,43,44]. The high standard morphological scores obtained in both sexes, with most animals classified as “superior” (mean total scores of 86.5 in males and 90.2 in females), reflect the rigorous application of the breed standard despite the limited census size. The residual variability observed in comb, wattles, ear lobes and plumage coloration, particularly in males, is consistent with the dynamics reported for other recovering local populations, in which chromatic stabilization often lags behind structural morphological traits [45,46]. Compared with other Spanish local breeds such as Castellana Negra or Utrerana, the Murciana shows marked sexual dimorphism that is already evident at early ages. From a morphological perspective, similarities can be observed between Murciana and Mallorquina (“Paja” variety) males, as well as between Murciana and Ibicenca (“Trigueña Plateada” variety) females, particularly regarding light plumage tones and pigment distribution on the back and tail [33,43].

Morphometric traits recorded in the Murciana chicken breed fall within the range reported for European local and Mediterranean chicken populations. In the present study, Murciana breeder males reached a mean BW of approximately 3.2 kg and a TC of 40.6 cm, whereas adult females averaged 2.4 kg and 35.2 cm, respectively. Breeders’ BW, TC and body measurements are comparable to those described for Italian and Portuguese slow-growing breeds, where adult males typically range between 2.5 and 3.5 kg and females between 1.8 and 2.5 kg [40,47,48]. These data are particularly relevant for refining breed standards, identifying animals deviating from the typical phenotype and supporting selection and management strategies that preserve breed identity while maintaining functional productivity.

### 4.3. Growth Performance

The growth trajectory of the Murciana breed showed clear sex-related differences in both magnitude and temporal dynamics. Males consistently exhibited higher BW throughout the experimental period and maintained greater ADG, particularly during the intermediate growth phase (28–84 days). In both sexes, ADG increased progressively up to approximately 42–56 days of age, followed by a stabilization phase and a subsequent decline. This pattern is consistent with growth dynamics reported in other slow-growing local chicken breeds, characterized by an early acceleration phase followed by a gradual reduction in growth intensity [48,49]. The Gompertz model adequately described these dynamics and confirmed the observed sexual dimorphism. Males exhibited higher BWa and MGR, together with a later Tip, indicating a more prolonged period of rapid growth. In contrast, females showed higher k values and reached the ip earlier, reflecting a more precocious growth trajectory. The estimated k values (0.020–0.023 d^−1^) fall within the range reported for Mediterranean local chicken breeds (≈0.017–0.021 d^−1^), with male values closely matching those previously described and female values being higher than the reported interval [36,47,48,50]. These differences are consistent with the corresponding variation in Tip, with males showing slightly delayed inflection ages (70.4 d) and females earlier ones (58.2 d). The divergence observed between final BW and the BWa estimated by the model, particularly in females, likely reflects that the growth curve was fitted within a pre-maturity window. In local breeds, extending the observation period beyond 180 days has been shown to improve the accuracy of asymptotic parameter estimation [40,47,48]. Despite this limitation, the model reliably captures the overall growth trajectory and relative differences between sexes. From an applied perspective, the combination of moderate growth rates (ADG ≈ 20 g/d in males and ≈13 g/d in females) and the relatively late inflection point in males positions the Murciana breed within the spectrum of slow-growing, dual-purpose genotypes.

### 4.4. Reproductive Performance, Egg Production, and Carcass Traits

Fertility and hatchability values observed in the Murciana breed fell within the medium and upper ranges reported for Mediterranean local chickens, respectively. Fertility rates of 80–95% and hatchability around 70–80% are commonly described under conservation-oriented and small-scale management systems [41,51]. These results indicate that the reproductive management protocols applied were adequate to maintain embryonic viability in a small-size conserved population, in line with FAO recommendations [23,34].

Egg production in Murciana hens reflected the combined effects of flock structure and environmental variability. The annual production value (116.6 eggs/hen/year) exhibited lower laying performance than commercial genotypes due to the absence of intensive selection [52]. However, this estimate primarily represents system-level performance and was influenced by continuous flock renewal and heterogeneous age structure. To the best of our knowledge, annual egg production data for Mediterranean local chicken breeds are scarce. In addition, seasonal variation in HDEP followed the expected pattern for non-controlled systems, with higher production in spring and a decline during summer, consistent with the effects of temperature and photoperiod on laying activity [53,54]. Most studies report laying performance in hens up to one year of age, whereas the present study included hens up to two years old. Peak HDEP reached approximately 50 and 46% in both one- and two-year-old hens, occurring during the spring period (16th–30th April). These values were below those reported for the Mugellese breed under natural management conditions (≈66%), while remaining within the broader range described for other Mediterranean local breeds (≈50–61%) in controlled management systems [41,51,55]. In our case, older hens maintained similar HDEP compared to younger individuals until the end of the spring season, after which an age-related decline was observed. This pattern suggests that two-year-old hens may still contribute effectively to reproductive performance during the main laying period, allowing partial flock renewal rather than complete annual replacement. Furthermore, the relatively high variability observed among pens highlights the heterogeneity inherent to conservation systems and reinforces the need to interpret average values with caution. Rather than reflecting inconsistent performance, this variability is characteristic of low-input systems, where productivity emerges from the interaction between genetic potential and environmental conditions.

The carcass profile of Murciana males was characterized by low to intermediate carcass yields, a clear predominance of leg quarters, and a moderate breast development. The BCY and NCY (77.6% and 61.9%, respectively) were broadly consistent with those reported for males of Portuguese local breeds slaughtered at older ages, although net carcass yield remained approximately 10% lower [10,56]. Similarly, values were within the lower values of the eviscerated carcass yield range (≈74–83%) described for other Mediterranean breeds slaughtered at somewhat later ages [37,57,58,59]. Leg quarters represented 29.3% of BCW and 36.7% of NCW, whereas breast yield accounted for between 13.5 and 16.9% of BCW. This distribution contrasts with modern commercial broiler lines, which are strongly selected for breast muscle deposition [1], but closely resembles the carcass composition described for European slow-growing local breeds such as Amarela, Preta Lusitânica, and Mos [38,40].

Taken together, these results indicate that the Murciana breed exhibits a balanced productive profile, combining stable reproductive performance with carcass traits characteristic of non-selected dual-purpose genotypes. This functional balance, rather than maximum productivity, represents a key attribute for its sustainable use within conservation-oriented and alternative production systems.

### 4.5. Limitations and Future Research Directions

Egg quality was not included in the present analysis and should be addressed in future studies to complement the characterization of the breed’s laying aptitude and its potential for product differentiation. Moreover, the absence of feed intake data precluded the evaluation of feed efficiency, which remains a key parameter for comparing productive performance across genotypes and production systems.

Future research should therefore focus on extending reproductive records across multiple laying cycles, incorporating egg quality traits, and integrating efficiency-related indicators, completing the evaluation of the breed and supporting its sustainable use within conservation-oriented production systems.

## 5. Conclusions

Our results fill a knowledge gap for a previously under-documented Spanish local breed and establish a solid baseline for its ongoing management and conservation status.

Despite its reduced population size, the Murciana chicken breed currently shows signs of demographic stabilization supported by a structured conservation program that has allowed the maintenance of a well-defined breed phenotype and high conformity to the official standard. From a productive standpoint, the breed displays reproductive efficiency, growth dynamics, carcass characteristics, and egg production patterns consistent with slow-growing Mediterranean dual-purpose genotypes adapted to extensive systems.

Overall, the Murciana chicken breed can be regarded as a viable local genetic resource with clear potential for conservation through use. Its long-term sustainability will depend on continued genetic monitoring, the extension of productive performance analysis over time in Mediterranean environments, and the reinforcement of valorization strategies linked to differentiated markets. In this context, the present study provides a reference framework to guide future conservation actions, support evidence-based decision-making within the breeding program, and facilitate the integration of the Murciana breed into sustainable agri-food systems while safeguarding its breed identity.

## Figures and Tables

**Table 1:** Characteristics of Murciana chicken breed farmers and their facilities in 2024.

Category		Quantity
Farmers	Total	37
	Men	34
	Women	3
Registration status	General Registry of Livestock Farms (REGA, Spain)	6
	“100% Murciana Autochthonous Breed” quality label	1
Production purpose	Self-consumption	35
	Commercial	1
	Breeding (multiplier)	1

**Table 2:** Standard morphological evaluation of Murciana growing chicken males and females (mean ± SD).

Parameter	Males (*n* = 71)	Females (*n* = 54)
Head (×2)	9.86 ± 0.35	9.93 ± 0.37
Tail (×1)	9.88 ± 0.38	10.0 ± 0.00
Chest, abdomen and back (×1)	8.79 ± 1.19	9.59 ± 1.05
Thighs, shanks and toes (×1)	9.29 ± 1.66	9.66 ± 1.11
Comb, wattles and ear lobes (×1)	7.46 ± 1.12	8.42 ± 0.81
Color (×4)	7.84 ± 0.98	8.17 ± 0.98
Total score	86.5 ± 4.45	90.2 ± 3.93

×1, ×2, ×4 indicate the weighting factor applied to each trait in the total score.

**Table 3:** Morphometric traits of Murciana chickens according to sex and age group (mean ± SD).

Parameter	Growing Females (*n* = 54)	Growing Males (*n* = 71)	Breeder Females (*n* = 45)	Breeder Males (*n* = 12)
Body weight (BW), g	1820 ± 139	2717 ± 242	2448 ± 289	3197 ± 306
Body length (BL), cm	41.0 ± 0.9	45.8 ± 1.9	41.7 ± 1.5	45.9 ± 1.9
Thoracic circumference (TC), cm	33.9 ± 1.1	39.0 ± 2.2	35.2 ± 1.8	40.6 ± 2.4
Tarsus diameter (TD), cm	4.05 ± 0.14	4.97 ± 0.24	4.11 ± 0.18	5.42 ± 0.40
Tarsus length (TL), cm	8.95 ± 0.29	11.1 ± 0.58	8.94 ± 0.37	10.8 ± 0.51
Wingspan (WS), cm	51.1 ± 1.8	59.9 ± 3.2	51.2 ± 2.7	58.2 ± 2.4
Wing length (WL), cm	20.3 ± 0.9	23.0 ± 1.0	20.2 ± 0.7	23.1 ± 1.1

Growing birds were 133 days old, whereas breeder females and males had ages of 392 and 660 days old in average, respectively.

**Table 4:** Observed body weight (BW) and average daily gain (ADG) of Murciana growing chicken males and females (mean ± SD).

	Males (*n* = 71)	Females (*n* = 54)
Age (d)	BW (g)	
0	43.9 ± 3.40	43.8 ± 4.21
14	146 ± 12.7	133 ± 11.3
28	359 ± 41.9	295 ± 27.2
42	646 ± 82.8	515 ± 54.6
56	1001 ± 117	804 ± 97.3
70	1354 ± 145	1043 ± 130
84	1713 ± 176	1274 ± 148
98	2027 ± 229	1460 ± 195
112	2362 ± 237	1643 ± 189
126	2655 ± 249	1772 ± 217
Period (d)	ADG (g/d)	
0–14	7.38 ± 0.81	6.40 ± 0.86
14–28	15.1 ± 2.87	11.7 ± 1.98
28–42	20.6 ± 3.34	15.7 ± 2.39
42–56	26.1 ± 3.87	20.4 ± 3.61
56–70	24.6 ± 2.52	16.8 ± 2.73
70–84	25.4 ± 3.48	17.3 ± 2.94
84–98	22.9 ± 5.63	12.6 ± 4.55
98–112	23.9 ± 6.49	13.2 ± 5.14
112–126	20.4 ± 3.50	9.53 ± 3.39
0–126	20.2 ± 1.93	13.4 ± 2.06

**Table 5:** Gompertz growth model parameters and derived traits for Murciana chicken breed males and females.

Parameter	Males (*n* = 71)	Females (*n* = 54)
Asymptotic body weight (BWa), g	3704	2317
b parameter	4.11	3.86
Relative growth rate (k), d^−1^	0.020	0.023
Age at inflection point (Tip), d	70.4	58.2
Body weight at inflection point (BWip), g	1363	852
Maximum growth rate (MGR), g/d	27.4	19.8

**Table 6:** Carcass characteristics of Murciana growing chicken males (mean ± SD, *n* = 20).

Parameter		Parameter	
Slaughter weight (SW), g	2730 ± 143		
*Carcass composition (weight)*		*Organs*	
Brute carcass weight (BCW), g	2117 ± 115.4	Gizzard, g	54.4 ± 11.9
Net carcass weight (NCW), g	1688 ± 95.9	Liver, g	48.5 ± 9.37
Breast *, g	286 ± 29.7	Heart, g	15.5 ± 1.62
Breast skin, g	37.1 ± 7.49	Gizzard, % BCW	2.57 ± 0.55
Leg quarters, g	620 ± 50.1	Liver, % BCW	2.29 ± 0.43
Wing, g	209 ± 12.4	Heart, % BCW	0.73 ± 0.07
Carcass frame, g	504 ± 39.2		
*Carcass composition (% of SW or BCW/NCW)*		*Non-edible parts*	
Brute carcass yield (BCY), % SW	77.6 ± 2.50	Feet, g	104 ± 9.30
Net carcass yield (NCY), % SW	61.9 ± 2.57	Head, g	90.1 ± 14.7
Breast, % BCW	13.5 ± 1.06	Neck, g	113 ± 22.9
Breast, % NCW	16.9 ± 1.33	Abdominal fat, % SW	1.20 ± 0.87
Leg quarters, % BCW	29.3 ± 1.51	Feet, % BCW	4.90 ± 0.39
Leg quarters, % NCW	36.7 ± 1.86	Head, % BCW	4.26 ± 0.69
Wing, % BCW	9.88 ± 0.62	Neck, % BCW	5.52 ± 1.07
Wing, % NCW	12.4 ± 0.78		
Carcass frame, % BCW	23.8 ± 1.67		
Carcass frame, % NCW	29.9 ± 2.03		

* Skinless and boneless.

## Data Availability

The relevant data are included within the article. Extra-data can be obtained from the corresponding author upon reasonable request.
