# Correction: Martínez-Martínez et al. Characterization of a Recovered Mediterranean Chicken Breed: The Case of Murciana. *Animals* 2026, *16*, 1793

**DOI:** 10.3390/ani16152279

**Published:** 2026-07-23

**Authors:** Laura Martínez-Martínez, Achille Schiavone, Juan Orengo, Eva Armero

**Affiliations:** 1 Department of Agricultural Engineering, Universidad Politécnica de Cartagena Member of European University of Technology EUT+, Paseo Alfonso XIII 48, 30203 Cartagena, Spain; achille.schiavone@unito.it (A.S.); eva.armero@upct.es (E.A.); 2 Department of Veterinary Sciences, University of Turin, Largo Braccini 2, 10095 Grugliasco, TO, Italy; 3 Department of Animal Production, Faculty of Veterinary Science, University of Murcia, 30100 Murcia, Spain; jorengo@um.es

**Funding Correction**

In the original publication [1], there was an error regarding the funding grant number. The correct funding information appears below:

This research was funded by Regional Government of the Region of Murcia, grant number FS/10.13039/100007801 22627/PI/24. España.

**Additional Affiliation**

In the published publication, there was an error regarding the affiliation for Juan Orengo. In addition to current affiliations, the additional affiliation should be as follows: Department of Animal Production, Faculty of Veterinary Science, University of Murcia, 30100 Murcia, Spain.

**Addition of an Author**

Juan Orengo was not included as an author in the original publication. The corrected Author Contributions statement appears here.

Conceptualization, L.M.-M., A.S. and E.A.; methodology, L.M.-M., J.O. and E.A.; validation, L.M.-M. and E.A.; formal analysis, L.M.-M. and E.A.; investigation, L.M.-M. and E.A.; resources, E.A.; data curation, L.M.-M.; writing—original draft preparation, L.M.-M.; writing—review and editing, L.M.-M., A.S., J.O. and E.A.; supervision, J.O. and E.A.; funding acquisition, J.O. and E.A. All authors have read and agreed to the published version of the manuscript.

The authors state that the scientific conclusions are unaffected. This correction was approved by the Academic Editor. The original publication has also been updated.
